# Diagnostic Value of Single-Photon Emission Tomography Stress Test in Patients With Suspected Coronary Artery Disease in Saudi Arabia

**DOI:** 10.7759/cureus.19071

**Published:** 2021-10-27

**Authors:** Mohammed S Alqarni, Ziad M Bukhari, Abdulkarim W Abukhodair, Dina Y Binammar, Atif Alzahrani, Abdulkareem Alkahtani, Saad Albugami

**Affiliations:** 1 College of Medicine, King Saud Bin Abdulaziz University for Health Sciences, Jeddah, SAU; 2 Medicine, King Abdullah International Medical Research Center, Jeddah, SAU; 3 Cardiac Sciences, Ministry of National Guard Health Affairs, Jeddah, SAU; 4 Medical Imaging, Ministry of National Guard Health Affairs, Jeddah, SAU; 5 Cardiology, King Faisal Cardiac Center, King Abdulaziz Medical City, Jeddah, SAU; 6 Cardiology, King Abdullah International Medical Research Center, Jeddah, SAU; 7 Cardiology, King Saud Bin Abdulaziz University for Health Sciences, Jeddah, SAU

**Keywords:** chronic stable angina, coronary artery disease, myocardial perfusion imaging, single-photon emission computed tomography, percutaneous coronary intervention

## Abstract

Background

Invasive coronary angiography (ICA) is the gold standard procedure for the diagnosis of coronary artery disease (CAD). ICA allows for clear visualization of the coronary arterial blood flow. Single-photon emission computed tomography (SPECT) is currently in widespread use to non-invasively evaluate patients known or suspected of coronary artery disease (CAD). This study aimed to examine the association between (SPECT) stress test and elective ICA in terms of diagnostic value in patients suspected of coronary artery disease at the King Faisal Cardiac Center (KFCC), Jeddah, Saudi Arabia.

Methods

This study is a retrospective diagnostic validation study using a consecutive sampling technique to select the study sample at KFCC. The study included all patients who presented with chest pain that were investigated with either exercise or pharmacologic myocardial perfusion SPECT study followed by elective ICA within six months from January 2015 to January 2020.

Results

A total of 207 patients met the inclusion criteria, where 43% (n = 90) of patients were females and 57% (n = 117) were males; 68% (n = 141) of the patients had both test results concordant (both SPECT and ICA results were in agreement). In 32% of the patients (n = 66), there was a discordant result (discrepant result between SPECT and ICA). SPECT had a sensitivity of 92.4% and a specificity of 26.3%. SPECT had a negative predictive value of 0.68 and a positive predictive value of 0.66 compared to ICA. There was a low degree of reliability between SPECT and ICA.

Conclusion

Reliability between the SPECT and ICA in exclusion of significant CAD is high. The rate of false-positive tests was high while the accuracy of SPECT in detecting CAD in patients with diabetes and hypertension was high. The overall reliability of SPECT to ICA in the Saudi population was low.

## Introduction

The World Health Organization reported coronary artery disease (CAD) as one of the major causes of mortality, killing around seven million people annually [1]. The overall prevalence of CAD in Saudi Arabia is estimated to be 5.5% [2]. Therefore the effective diagnosis of CAD is required using safe and accurate diagnostic methods to risk stratify patients appropriately. Nowadays, invasive coronary angiography (ICA) is the procedure of choice for an accurate diagnosis of CAD. ICA allows for clear visualization of the coronary arterial blood flow, which helps to diagnose and risk stratify patients and provide a mode for delivering therapy [3]. However, ICA is occasionally not easily accessible and not widely accepted by many patients as the majority prefers non-invasive diagnostic tests [4]. ICA is an invasive procedure but not without complications, such as acute kidney injury, stroke, arrhythmia, and a mortality rate of 0.45% [5,6]. 

Radionuclide cardiac stress testing with its two techniques, positron emission tomography (PET) and single-photon emission computed tomography (SPECT), is currently in use to evaluate patients known or suspected to have CAD. In addition, they have a superior utility in evaluating patients with left bundle branch block, ventricular paced rhythm, and left ventricular hypertrophy. Studies have linked SPECT with higher false-positive results when used to evaluate CAD patients due to limited spatial resolution in certain cases (e.g., breast attenuation, left ventricular hypertrophy) [7-9]. The use of the nuclear stress test is relatively safe, most of the side effects are minimal and transient, and the majority is linked to the pharmacological stressors used. However, two case series studies have reported coronary vasospasm as a rare adverse outcome occasionally occurring during or after an adenosine stress test [10]. 

Although ICA is considered the gold standard for detecting clinically significant CAD, the new generation high-speed (HS)-SPECT provides high image quality and similar overall diagnostic accuracy to ICA. Combined supine and prone stress imaging provided the best diagnostic accuracy [11-13]. Previous studies have shown that the degree of concordance between SPECT and ICA ranges between 60% and 90% [14]. Our study aimed to determine the efficacy of SPECT in assessing the detection of CAD by comparing the results to the gold standard ICA and correlating results to patient demographics, co-morbidities, prognosis, and laboratory results at the King Faisal Cardiac Center, Jeddah, Saudi Arabia.

## Materials and methods

This is a retrospective diagnostic validation study using a non-probability consecutive sampling technique to select the study population at King Faisal Cardiac Center, Jeddah, Saudi Arabia. The research project was approved by King Abdullah International Medical Research Center, Jeddah, Saudi Arabia (#IRBC/1293/20). 

The study included all patients who presented with chest pain suggestive of stable angina who underwent exercise or pharmacologic myocardial perfusion SPECT study followed by elective ICA within six months period. The data were collected from January 2015 till January 2020. The exclusion criteria included patients younger than 18 years old, patients who required an emergency ICA due to the development of unstable angina, non-ST-elevation myocardial infarction (NSTEMI), or ST-elevation myocardial infarction (STEMI), and patients with a history of prior coronary revascularization. A positive case of CAD on ICA was considered when there was significant coronary artery stenosis with a value of ≥70% luminal diameter narrowing in the left coronary system and ≥50% luminal diameter narrowing in the case of right coronary artery. SPECT study was reported to be abnormal based on the readings of three experienced nuclear imaging consultants. Data were collected using the patient health electronic system. Patient’s demographics, past medical history, laboratory values, ICA and SPECT-related data, echocardiography, ECG data at the time of SPECT, and follow-ups were collected.

For the analysis, categorical variables were summarized by frequencies and percentages, and continuous variables by means and standard deviations, or medians and interquartile ranges if their distributions were skewed. Cronbach’s alpha test was used to assess the reliability between SPECT and ICA. The correlation between the SPECT and ICA was compared with different variables using chi-square test, Wilcoxon rank-sum test, or Fisher's exact test. After dividing the study population into four groups based on the outcomes of the SPECT and ICA, they were compared based on the different study variables using Fisher's exact test or Kruskal-Wallis test. Variables with a p-value less than 0.05 were considered significant. All statistical analyses and assessments of the model’s performance were conducted using the R software, version 3.3.0 (R Foundation for Statistical Computing, Vienna, Austria) [15].

## Results

**Figure 1:**
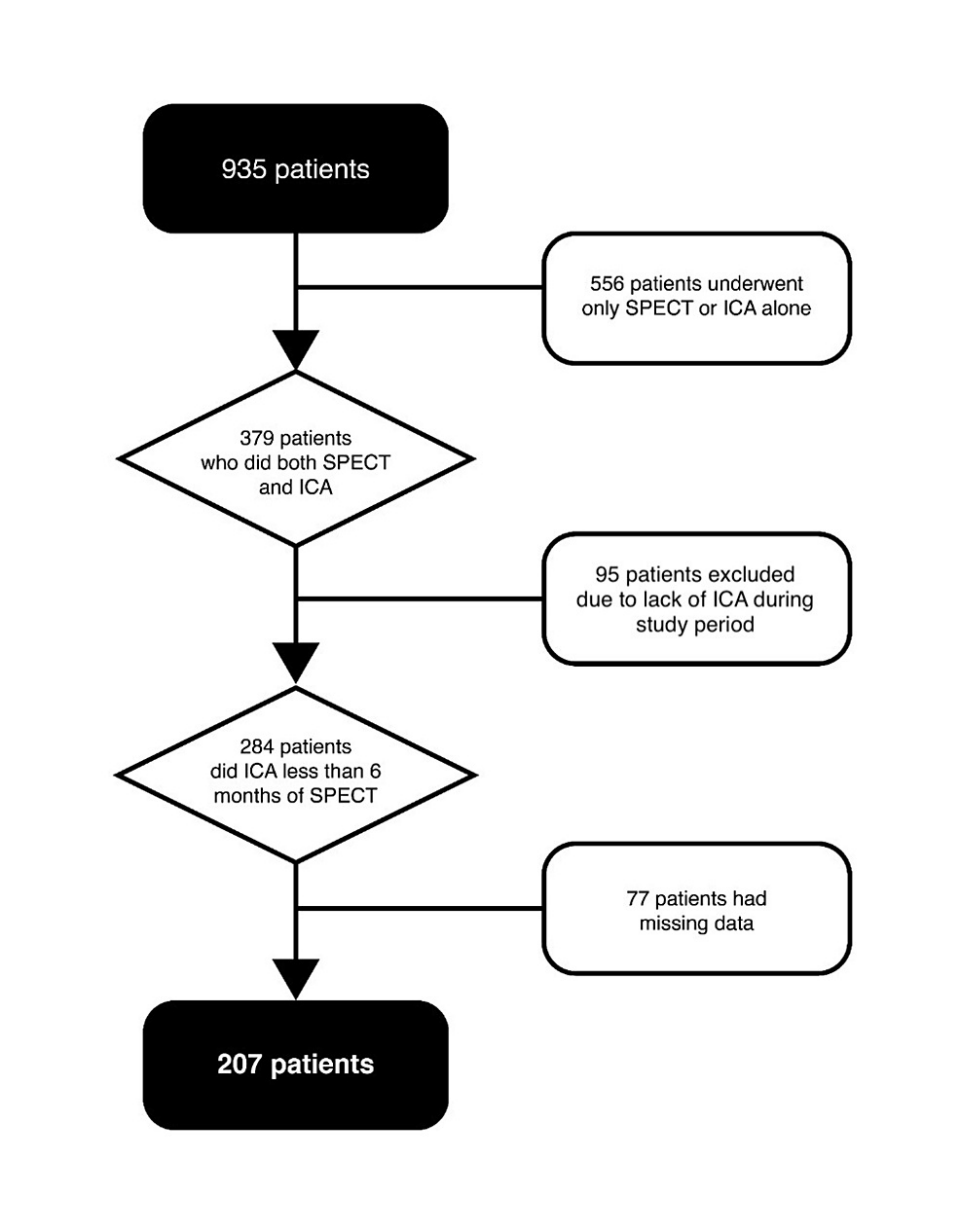
The flowchart of the inclusion and exclusion criteria ICA: invasive coronary angiography; SPECT: single-photon emission computerized tomography

A total of 935 patients were reviewed who had ICA and SPECT within the study period, only 207 patients have met the inclusion and exclusion criteria; the flowchart is shown in Figure 1. Of the study population, 43% (n = 90) were females and 57% (n = 117) were males. The mean age of the study population was 65 ± 11 years; 77% (n=159) were diabetic, 78% (n=162) were hypertensive, and 65% (n=135) were suffering from dyslipidemia. The median (IQR) glycated hemoglobin (HbA1c) of the whole population was 7.3 (6.3-8.5). The patients were further divided into two groups based on agreement or discrepancy of the SPECT with ICA results; 68% (n = 141) patients were in the agreement group, with 121 patients having positive results in both test modalities (true-positive rate of 58.5%) and 20 patients had negative results in both modalities (true-negative rate of 9.7%). There were 66 (32%) patients in the discrepant group, most (n = 56) of them had a positive SPECT followed by a negative ICA with a false-positive rate of 27%, and only 10 patients had a negative SPECT with positive ICA results with a false-negative rate of 4.8% as shown in Table 1.

**Table 1:** ICA and SPECT results for the whole study population. ICA: invasive coronary angiography; SPECT: single-photon emission computerized tomography

		ICA		
	Result	Negative	Positive	Total
SPECT	Negative	20	10	30
	Positive	56	121	177
	Total	76	131	207

Using Cronbach’s alpha test, the reliability between SPECT and ICA was measured to be 0.4, indicating a low level of agreement. In comparing SPECT results to ICA, SPECT had a sensitivity of 92.4% and a specificity of 26.3%. SPECT had a negative predictive value of 0.68 and a positive predictive value of 0.66 compared to ICA. The demographics, co-morbidities, and laboratory results compared between the two groups (concordant and discrepant groups) are shown in Table 2.

**Table 2:** Demographics, comorbidities, and laboratory results in discrepant and agreement groups. *Statistical tests performed - chi-square test of independence, Wilcoxon rank-sum test, and Fisher's exact test. BMI: body mass index; DM: diabetes mellitus; HTN: hypertension; DYS: dyslipidemia; CKD: chronic kidney disease; HF: heart failure; HDL: high-density lipoprotein; LDL: low-density lipoprotein

Characteristic	Discrepancy, N = 66	Agreement, N = 141	p-Value*
Gender			0.041
Female	36 (55%)	54 (38%)	
Male	30 (45%)	87 (62%)	
Age			0.3
Mean (SD)	63 (12)	66 (11)	
BMI			0.14
Mean (SD)	33 (8)	32 (10)	
Smoking			>0.9
No	58 (88%)	125 (89%)	
Yes	8 (12%)	16 (11%)	
DM			0.029
No	22 (33%)	26 (18%)	
Yes	44 (67%)	115 (82%)	
HTN			0.13
No	19 (29%)	26 (18%)	
Yes	47 (71%)	115 (82%)	
DYS			0.6
No	21 (32%)	51 (36%)	
Yes	45 (68%)	90 (64%)	
CKD			>0.9
No	65 (98%)	136 (97%)	
Yes	1 (1.5%)	4 (2.9%)	
Unknown	0	1	
HF			>0.9
No	45 (68%)	95 (67%)	
Yes	21 (32%)	46 (33%)	
HbA1c			<0.001
Median (IQR)	6.50 (5.80, 7.40)	7.60 (6.50, 8.70)	
Creatinine			0.3
Median (IQR)	78 (66, 96)	81 (69, 104)	
HDL			0.12
Median (IQR)	1.00 (0.84, 1.19)	0.95 (0.80, 1.06)	
LDL			0.4
Median (IQR)	2.48 (1.85, 2.96)	2.33 (1.81, 2.92)	

Patients were further subdivided into four groups based on the results of the SPECT and ICA demographics as shown in Table 3. The median (IQR) of total ischemia scar was 9 (2-14) and the mean of total scar size was 0.47 ± 2.01. Other characteristics of the SPECT study are shown in Table 4.

**Table 3:** Demographics, comorbidities, and laboratory results compared in the four groups of the study population. *Statistical tests performed - Fisher's exact test and Kruskal-Wallis test. ICA: invasive coronary angiography; SPECT: single-photon emission computerized tomography; BMI: body mass index; DM: diabetes mellitus; HTN: hypertension; DYS: dyslipidemia; HDL: high-density lipoprotein; LDL: low-density lipoprotein

Characteristic	ICA negative, SPECT positive, N = 56	ICA positive, SPECT negative, N = 10	ICA and SPECT negative, N = 20	ICA and SPECT positive, N = 121	p-Value*
Gender					0.002
Female	31 (55%)	5 (50%)	14 (70%)	40 (33%)	
Male	25 (45%)	5 (50%)	6 (30%)	81 (67%)	
Age					0.2
Mean (SD)	64 (12)	61 (10)	62 (9)	66 (11)	
BMI					0.012
Mean (SD)	34 (8)	27 (4)	32 (6)	32 (10)	
Smoking					0.7
No	50 (89%)	8 (80%)	17 (85%)	108 (89%)	
Yes	6 (11%)	2 (20%)	3 (15%)	13 (11%)	
DM					0.001
No	18 (32%)	4 (40%)	9 (45%)	17 (14%)	
Yes	38 (68%)	6 (60%)	11 (55%)	104 (86%)	
HTN					0.018
No	14 (25%)	5 (50%)	7 (35%)	19 (16%)	
Yes	42 (75%)	5 (50%)	13 (65%)	102 (84%)	
DYS					>0.9
No	18 (32%)	3 (30%)	7 (35%)	44 (36%)	
Yes	38 (68%)	7 (70%)	13 (65%)	77 (64%)	
HbA1c					<0.001
Median (IQR)	6.55 (5.77, 7.70)	6.50 (6.08, 7.02)	6.50 (5.77, 7.58)	7.80 (6.70, 8.90)	
Creatinine					0.003
Median (IQR)	76 (65, 92)	82 (70, 161)	70 (64, 77)	85 (71, 107)	
HDL					0.002
Median (IQR)	1.00 (0.84, 1.19)	1.04 (0.89, 1.16)	1.12 (0.96, 1.41)	0.93 (0.79, 1.03)	
LDL					0.058
Median (IQR)	2.34 (1.78, 2.93)	2.66 (2.62, 3.32)	2.70 (2.08, 3.53)	2.31 (1.80, 2.77)	

**Table 4:** Characteristics of the SPECT studies for the study population. LV TID: left ventricle transient ischemic dilatation; LVEF: left ventricle ejection fraction; RV: right ventricle; LAD: left anterior descending artery; LCx: left circumflex; RCA: right coronary artery

Characteristic	N = 207
Total ischemia size (%)	
Median (IQR)	9 (2, 14)
Total scar size (%)	
Mean (SD)	0.47 (2.01)
LV TID	
Median (IQR)	1.03 (0.97, 1.12)
LVEF rest	
Median (IQR)	55 (43, 63)
Mean (SD)	51 (19)
LVEF stress	
Median (IQR)	55 (42, 64)
Mean (SD)	52 (17)
RV uptake	
No	200 (97%)
Yes	7 (3.4%)
Localization ischemia	
LAD	122 (59%)
LCx	13 (6.3%)
Multiple places	18 (8.7%)
None	27 (13%)
Other	4 (1.9%)
RCA	23 (11%)

The agreement of ischemic lesion location in SPECT and ICA was tested in the LAD with 11% sensitivity and 96% specificity. Localization to the right coronary artery and left circumflex was not compared between SPECT and ICA due to the low sample size. ECG abnormalities during the stress part of the SPECT study compared to the ICA result had a sensitivity of 62% and a specificity of 33%. Mortality was detected in six patients with a rate of 2.9%.

## Discussion

In this retrospective study involving a small but very well-defined group of patients, we assessed SPECT to detect significant CAD compared to ICA as the reference standard. The reliability was low between the SPECT and ICA; the Cronbach’s alpha test was measured to be 0.4. Our study sensitivity was acceptable at 92.4% compared to what previous studies have reported (88.5-94%), while our study specificity was found to be 26.3%. This contrasts with what has been previously reported to be the specificity of SPECT for the detection of CAD (86-89.3%) [14,15]. We think this may likely be due to several factors such as the possibility of interobserver variability between the three readers. Lack of blindness and standardization of the reading protocols in addition to the observational nature of the study. The negative predictive value of our study was 68% and a positive predictive value of 66%. This is again in contrast to what has already been reported by previous international studies which reported a negative predictive value of 80% and the positive predictive value of 93% [5]. The ECG changes during the stress part of the SPECT had low sensitivity and specificity for detection of CAD when compared to ICA findings in concordance to the literature [16,17].

Females were significantly more likely to have a discrepant false-positive result (p = 0.002) contrary to what was previously reported with SPECT in women having higher accuracy for detecting CAD [18,19]. This highlights the need to improve the diagnostic accuracy through the adoption of measures that correct attenuation artifact through a combination of (1) supine and prone imaging, (2) supine and upright imaging, or (3) direct correction using either line sources or computed tomography (CT) [18]. 

Our study demonstrated the significant reliability of the correlation between SPECT and ICA in patients with diabetes mellitus in the agreement group with a p-value of 0.029, which is similar to what was reported by others; however, Kang et al. have compared sensitivity, specificity, and accuracy of SPECT in patients with and without diabetes mellitus and found no significant difference in the sensitivity and specificity of SPECT in the two groups [20,21]. The sensitivity and specificity of SPECT for detecting CAD, with the criteria of 50% as significant stenosis were 86% and 56% in diabetics and 86% and 46% in non-diabetics with no significant difference noted statistically [21,22]. 

In addition, we found a significant concordance between positive SPECT and ICA (p = 0.018) among hypertensive patients when compared to those without hypertension. This contrasts with what Elhendy et al. have reported with no significant difference between patients with and without hypertension with respect to sensitivity, specificity, and accuracy for the overall and regional diagnosis of coronary artery disease [23]. 

Our study has shown a low degree of overall reliability and diagnostic accuracy between SPECT and ICA in King Faisal Cardiac Center (KFCC), Jeddah, Saudi Arabia. This has a significant impact on the clinical services rendered to its patients and calls for measures of quality assurance and improvement projects to be undertaken. The interobserver interpretive variability and study protocol standardization could account for possible bias in this study outcomes. We, therefore, are launching a larger and prospective study with the help of a core laboratory to test for these findings. In addition, coronary artery functional studies using the instant flow reserve (iFR), fractional flow reserve (FFR), or coronary flow reserve (CFR) were not performed in all patients in the catheterization laboratory which may play a role in the detection of possible microvascular angina in patients with normal epicardial coronary arteries. 

Limitations

The retrospective nature, single-center design, and limited sample size of our study limit generalizability. It has an inherent selection bias and lack of core laboratory can also lead to interpretive bias. Many patients were excluded due to incomplete clinical data and lack of elective ICA. Yet our data is representative of a well-defined group of patients, and it is the first to report on patterns of myocardial perfusion SPECT from Saudi Arabia.

## Conclusions

In conclusion, negative myocardial perfusion SPECT is exceptionally reliable for exclusion of significant coronary artery disease detected by ICA in the Saudi population. Positive SPECT however has a high false-positive rate. There is low overall reliability of SPECT compared to ICA. Concordance rate is significant among symptomatic patients with hypertension and diabetes mellitus.
